# Diagnostic value of cardiac miR-126-5p, miR-134-5p, and miR-499a-5p in coronary artery disease-induced sudden cardiac death

**DOI:** 10.3389/fcvm.2022.944317

**Published:** 2022-08-25

**Authors:** Linfeng Li, Xiangwang He, Min Liu, Libing Yun, Bin Cong

**Affiliations:** ^1^Department of Forensic Pathology, West China School of Basic Medical Sciences and Forensic Science, Sichuan University, Chengdu, China; ^2^Department of Forensic Medicine, Hebei Medical University, Shijiazhuang, China

**Keywords:** coronary artery disease, sudden cardiac death, microRNA, myocardial hypertrophy, myocardial fibrosis

## Abstract

**Background:**

The identification of coronary artery disease-induced sudden cardiac death (CAD-SCD) has always been a medical challenge. MicroRNAs (miRNAs) played vital roles in pathogenesis processes and served as potential biomarkers for cardiovascular and many other diseases. The aim of this study was to investigate the diagnostic value of the specific miRNAs for CAD-SCD.

**Methods:**

A total of 30 autopsy-verified CAD-SCD victims were selected, including 18 individuals who experienced more than once asymptomatic myocardial ischemia (CAD-activated SCD) and 12 victims without prominent pathological features of insufficient blood supply (CAD-silent SCD). Meanwhile, 30 traumatic victims were enrolled as controls. Systematic postmortem examinations were performed in all study population. The expressions of cardiac miR-126-5p, miR-134-5p, and miR-499a-5p were analyzed by real-time quantitative polymerase chain reaction (RT-qPCR).

**Results:**

RT-qPCR showed significant downregulations of miR-126-5p and miR-499a-5p in CAD-SCD victims, with no obvious difference in miR-134-5p. Receiver-operating characteristic analysis revealed the diagnostic performance of miR-126-5p (areas under the curve [AUC] = 0.76) and validated miR-499a-5p (AUC = 0.82) as a sensitive marker. Additionally, the decreased expression of the two specific cardio-miRNAs was detected for discriminating CAD-silent SCD and CAD-activated SCD. Compared with the limited diagnostic value of single miR-126-5p and miR-499a-5p, their combination could achieve better discriminative capacity (AUC = 0.82, sensitivity = 91.7%, specificity = 77.8%).

**Conclusion:**

Cardiac miR-126-5p and miR-499a-5p presented good diagnostic abilities for CAD-SCD, and their combination could help evaluate CAD condition. These targeted miRNAs as novel biomarkers are expected to be useful to discriminate the detailed causes in real SCD cases.

## Introduction

Sudden cardiac death (SCD) represents one of the most frequent types of cardiovascular deaths, causing a substantial public health burden. It contributes to a considerable number of unexpected natural deaths worldwide, especially for the young and middle-aged people (1). Coronary artery disease (CAD) is the leading cause of SCD, and CAD-induced SCD (CAD-SCD) is responsible for ~80% of unexpected cardiac deaths (2).

Due to its sudden and unexpected characteristics, the process of SCD is quite rapid, which means there is almost not enough time for specific clinical examinations to investigate the causes. Postmortem diagnosis of CAD-SCD is difficult for clinical and forensic pathologists. Although gross morphology and histology are sufficient for the identification of coronary atherosclerosis, there is very little evidence for the specificity of acute myocardial injury that correlated CAD to SCD because of the short duration of ischemia before cardiac arrest. Common myocardial markers, such as cardiac troponin T, are limited to be used at autopsy to identify cardiac damage (3, 4). Thus, the diagnosis of CAD-SCD not only needs a comprehensive autopsy, but also usually requires consideration of depictions of death from witnesses, medical history, and other death information. However, plenty of SCD victims lack the abovementioned information, representing a huge challenge in diagnosis.

In addition, evaluating the condition of CAD is a key element for the identification of CAD-SCD. Myocardial hypertrophy and fibrosis are representative manifestations of repeated hypoxia-ischemia, reflecting the alteration of cardiac function (5). These features are able to be observed under the microscope and used to assist in the estimation of anti-ischemic ability of the heart. If there is a lack of such changes in some CAD-SCD victims, it is difficult to interpret the cardiac function when blood supply is sharply reduced (6). Hence, it is important to find other indicators that could be used for the judgment of cardiac reserve functions.

MicroRNAs (miRNAs) are a type of noncoding single-stranded RNAs containing ~22 nucleotides. They play important regulatory roles by combining with the specific binding sites. Multiple miRNAs are cardiomyocyte-enriched, involved in maintaining cardiac structure and function, as well as moderators that participate in a variety of cardiovascular diseases, especially in ischemic heart diseases (7). Previous studies reported that a number of miRNAs were related to coronary atherosclerosis and acute heart attack (8). miR-126 is an important regulator in vascular homeostasis, exerting its effect on myocardial infarction (9). miR-134 plays a key role in cardiomyocyte apoptosis during acute cardiac ischemia (10). miR-499 was found to inhibit cardiomyocyte apoptosis and against myocardial injury (11). The three miRNAs could specifically change in acute coronary events and be considered as new clinical biomarkers to detect myocardial infarction and predict prognosis of patients (12). But the association between these miRNAs and CAD-SCD remains obscure, and their diagnostic value of SCD has not been well studied, needing further investigations.

In this study, we analyzed the expression of miR-126-5p, miR-134-5p, and miR-499a-5p in CAD-SCD victims using real-time quantitative polymerase chain reaction (RT-qPCR). The aim was to explore whether the specific miRNAs could be used to diagnose CAD-SCD and differentiate CAD condition at autopsy.

## Materials and methods

### Study populations

We selected 30 SCD victims with autopsy-verified CAD at the Department of Forensic Pathology of Sichuan University, and forensic examinations were performed between 2018 and 2020. The postmortem interval was no more than 48 h (7 days if corpses are frozen well), and no obvious sign of spoilage was found at autopsy. According to the proposal of the World Health Organization (13), the definition of SCD in this study was death within 1 h after the onset of first cardiac symptom, with witnesses. If there is no witness, deceased patients, who were seen to be alive and apparently healthy within 24 h before death, were also considered as SCD.

Comprehensive medico-legal examinations were performed on all victims, including autopsy, histological examination, regular toxicological analysis, and molecular pathological test if required until the cause of SCD was clarified. Left anterior descending (LAD), left circumflex (LCX), and right coronary artery (RCA), three major branches of coronary system, were separated carefully and cut at every 0.3 cm for inspection. If atherosclerotic plaques are found in the walls, the abnormal artery would be sent to microscopic examination. Lethal CAD was considered when the stenosis level of at least one major branch was >60%. Meticulous cardiac examinations were performed on all victims to rule out other fatal cardiovascular diseases, including myocarditis, valvular diseases, and cardiomyopathy. In this study, besides death being completely responsible for fatal CAD while other causes of death were excluded by postmortem investigation, CAD-SCD was diagnosed if at least one of the following signs was found: (1) unstable plaque (plaque rupture, thick fibrous cap, angiogenesis, etc.); (2) coronary embolism; (3) contraction band necrosis; (4) myocardial hemorrhage; (5) acute inflammatory cells infiltration; and (6) sudden cardiac symptoms within 1 h before death clarified by a witness.

Individuals who met any of the following conditions were excluded: (1) age < 18; (2) intoxication caused by any poison or drug; (3) signs of asphyxia; and (4) a history of cardiac surgery.

A total of 30 individuals who died of fatal trauma were included in the control group. All controls had serious CAD (degree of coronary stenosis was > 60%), but it played no role in their death. The fatal traumatic victims had the same distribution of age and gender as the CAD-SCD group.

This study was approved by the medical ethics committee of Sichuan University. Informed consent was obtained from the relatives of all study participants. All subjects enrolled in the study were genetically unrelated Chinese Han population.

### Pathological investigation and sample collection

The entire heart was taken out from the corpse, and meticulous inspections were performed before and after fixation in 10% formalin. Coronary arteries, left and right ventricles, ventricular septal, and all suspected abnormal cardiac tissues were routinely sent for histological examination. Myocardial hypertrophy and fibrosis, manifestations of unrecognized CAD activity, were judged through macroscopic and microscopic inspections by two independent senior forensic pathologists. According to the presence of myocardial hypertrophy and fibrosis, and other evidence of antecedent myocardial ischemia, the study population was divided into SCD victims with an activated CAD, who had experienced recurrent asymptomatic myocardial ischemia (CAD-activated SCD), and victims with a silent CAD, who were found to have no feature of old symptom-free myocardial anemia by regular pathological investigation (CAD-silent SCD).

Approximately 4 g myocardial tissue of left ventricular wall was collected for miRNAs quantitation. Formalin-fixed paraffin-embedded (FFPE) tissue blocks and hematoxylin and Eosin (HE) staining sections were made according to conventional methods.

### RNA extraction, reverse transcription, and RT-qPCR

Although the degradation and chemical modification of nucleic acid in FFPE tissue is an inevitable limitation, unlike DNA and mRNA, miRNA could resist postmortem decay and chemicals due to its characteristics of small molecules, which makes miRNA have a potential application in assisting the forensic diagnosis (14). Previous studies had successfully extracted miRNAs from postmortem FFPE tissues and conducted RT-qPCR analysis (15, 16). Total RNAs were extracted from FFPE blocks using miRNeasy FFPE Kit (QIAGEN, Hilden, Germany). For each FFPE sample, 2–3 sections of 10 μm thickness were cut from FFPE tissue block and transferred to a microcentrifuge tube. The rest of the steps of purification were carried out according to the handbook of the manufacturer. The total RNA concentration was determined using NanoDrop1000 (Thermo Fisher Scientific, Waltham, MA, USA). All RNA extracts were diluted to 5 ng/μl with RNase-free water. Reverse transcription was performed using miRCURY LNA RT Kit (QIAGEN, Hilden, Germany). The preparation of the reaction was completed on ice. RT-qPCR was performed in ABI 7500 RT-qPCR system (Thermo Fisher Scientific, Waltham, MA, USA) using miRCURY LNA SYBR^®^ Green PCR Kits (QIAGEN, Hilden, Germany) according to the instructions of the manufacturer. U6 snRNAs were used as the endogenous control for miRNA quantification.

### Statistical analysis

The relative expression of targeted miRNAs was calculated using the 2^−Δ*ΔCt*^ method (17). ΔCt is the difference between interested miRNA and endogenous control, calculated through ΔCt_miRNA_ = Ct_miRNA_- Ct_housekeeping_. ΔΔCt is the difference from the ΔCt_miRNA_ of control group, calculated through ΔΔCt_miRNA_ = ΔCt_miRNA_- ΔCt_miRNAofcontrolgroup_.

Continuous variables are expressed as mean ± standard deviation and compared by independent Student's *t*-test or rank-sum test, depending on the distribution and homogeneity of variance. The diagnosed powers of the targeted miRNAs were evaluated by receiver-operating characteristic (ROC) analysis, and area under the curve (AUC) was calculated. Statistical significance was considered when *p* < 0.05. All statistical analyses were performed in IBM SPSS Statistics 26.0 (IBM Corp, Armonk, NY, USA).

## Results

### Basic and morphological characteristics of the study population

The mean age of the CAD-SCD victims was 52.2 ± 9.3 years, and most of them were males (63.3 vs. 36.7%). Most victims died unexpectedly at night-time (56.6 vs. 43.4%). CAD-SCD was more popular in winter (12/30). All subjects tested negative for abused drugs and alcohol. The majority of the CAD-SCD victims had multiple-vessel coronary atherosclerosis. LAD and RCA were the most common abnormal branches in the SCD group, and the prevalence of RCA serious atherosclerosis in CAD-SCD victims had a statistical difference with the control group (70.0 vs. 43.3%, *p* = 0.037). Detailed results are described in Supplementary Table 1.

**Figure 1:**
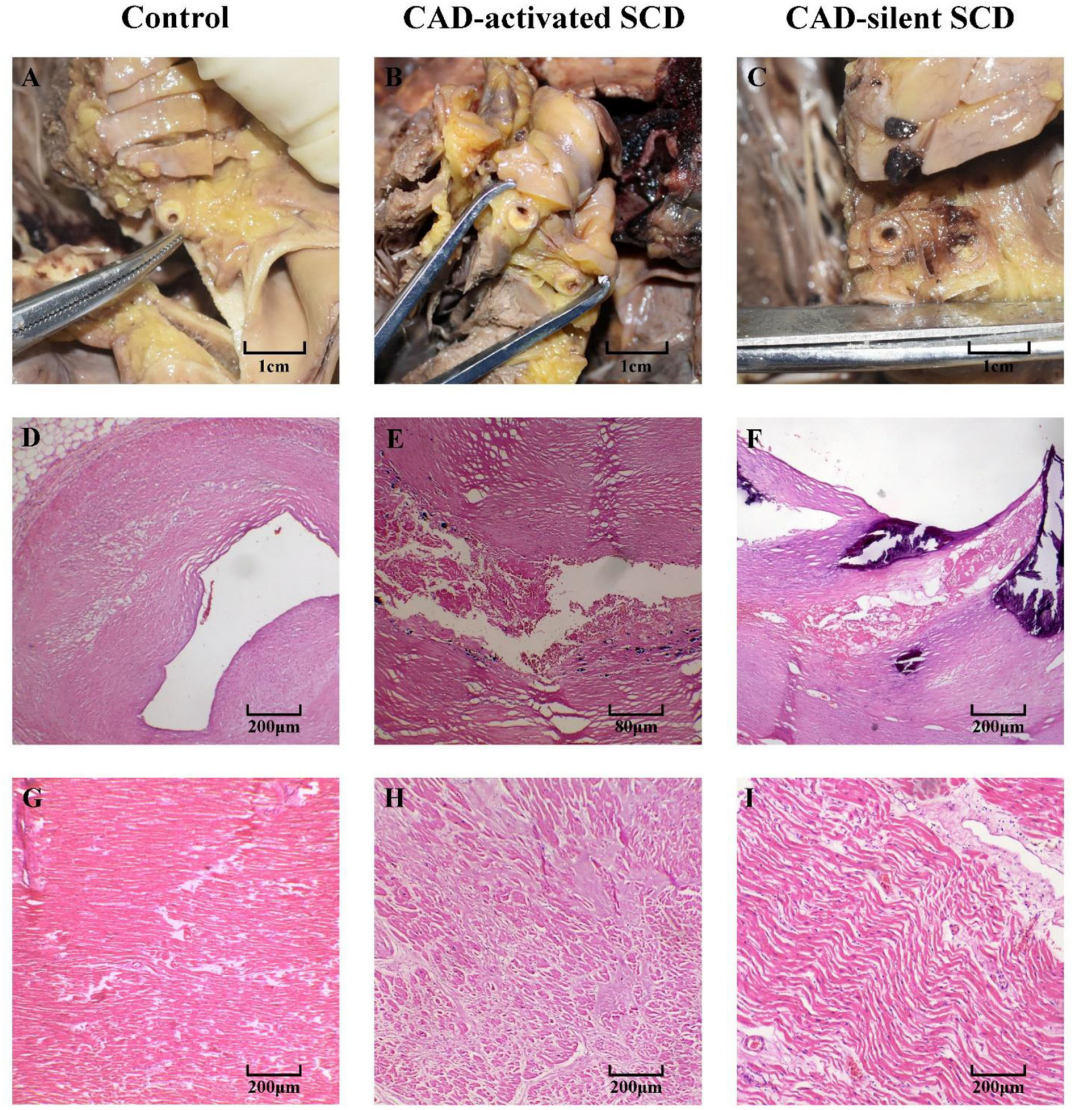
The cardiac pathological changes of CAD-activated SCD, CAD-silent SCD, and control. **(A–C)** Gross investigation of coronary arteries: atherosclerotic plaques caused significant luminal stenosis. Stable plaques were found in the control group **(D)**. CAD-activated SCD and CAD-silent SCD victims presented with unstable plaques, including rupture and hemorrhage **(E,F)**. Myocardial tissues of control subjects were basically normal, with mild interstitial expansion **(G)**. Myocardial hypertrophy and dense fibrotic scar were found in CAD-activated SCD victims **(H)**. Just slight pathological changes, including interstitial edema, wavy changes of cardiomyocytes, and inflammatory cell infiltration, were identified in CAD-silent SCD victims **(I)**.

In cardiac pathological examination, serious coronary atherosclerosis with obstructive effect was found in all subjects, which induced at least a 60% reduction in coronary blood flow (Figures 1A–C). In histology, the coronary atherosclerotic plaques of controls were stable, manifested by a well-developed fibrous cap and a modest lipid pool (Figure 1D). In contrast, complicated and vulnerable plaques, such as thin fibrous cap and calcified nodules, were commonly found in CAD-SCD victims, and the signs of acute events (i.e., plaque rupture and hemorrhage) were identified either (Figures 1E,F). There was little histological change in myocardial tissue except for a mild degree of hyperplasia of interstitial tissue in controls (Figure 1G). Myocardial hypertrophy and fibrosis were found in 18 CAD-SCD victims, labeling them as CAD-activated SCD victims. Their myocardial fibers were disordered and hypertrophied, with proliferated fibrous tissues and myocardial scars in the microscope (Figure 1H). Conversely, in CAD-silent SCD victims, only slight pathological lesions were identified, including interstitial edema, wavy changes of cardiomyocytes, and inflammatory cell infiltration (Figure 1I).

### The diagnostic value of miR-126-5p, miR-134-5p, and miR-499a-5p for CAD-SCD

After analyzing the expression of the three cardiac miRNAs, RT-qPCR revealed a downregulation of miR-126-5p in CAD-SCD victims by ~3.1-fold, with a significant difference. Additionally, the expression of miR-499a-5p was also remarkably downregulated by ~1.9-fold. However, there was no statistically significant trend in miR-134-5p between the two groups. The different levels of the miRNAs are shown in Supplementary Figure 1.

**Figure 2:**
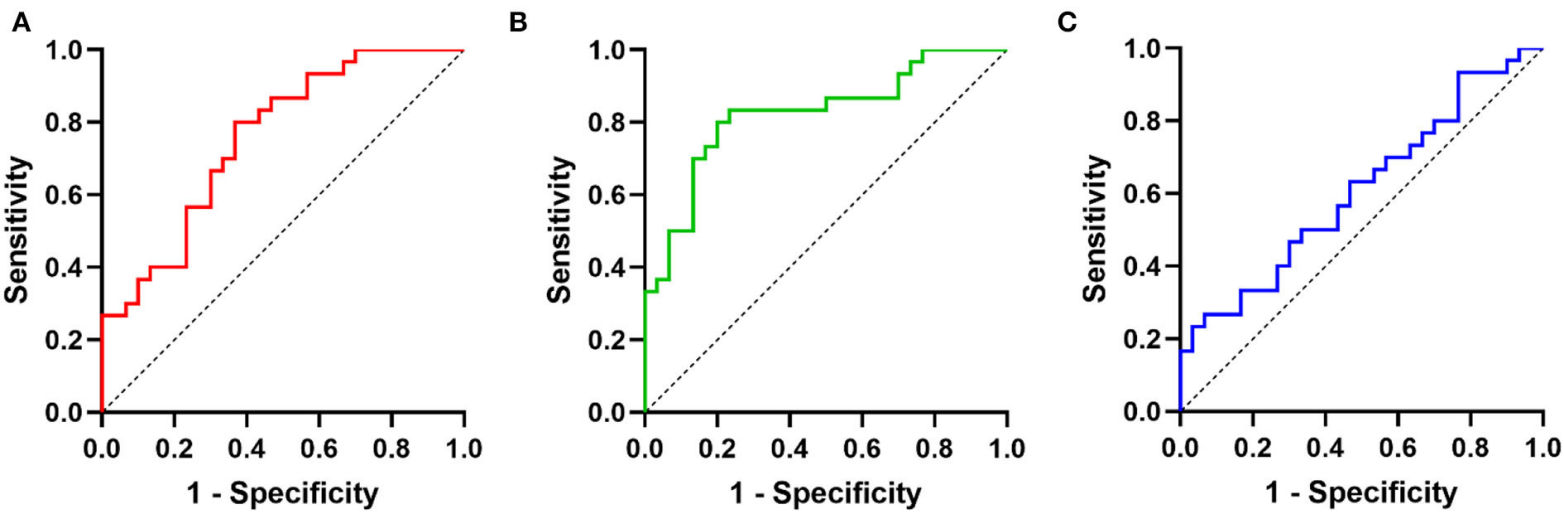
The diagnostic value of three miRNAs for CAD-SCD: ROCs of **(A)** miR-126-5p; **(B)** miR-499a-5p; **(C)** miR-134-5p.

ROC analysis was performed to evaluate the diagnostic powers of targeted miRNAs, and the results showed miR-126-5p and miR-499a-5p had discriminatory utility for CAD-SCD (Figure 2). The sensitivity and specificity of miR-126-5p were 80.0 and 63.3% at maximum Youden index. The AUC was 0.76 (95% confidence interval [CI] 0.64–0.88, *p* < 0.001). The sensitivity and specificity of miR-499a-5p were both 80.0% when Youden index was maximum. The AUC was 0.82 (95% CI = 0.72–0.93, *p* < 0.001). But the AUC of miR-134-5p was 0.61 (*p* > 0.05), with no diagnostic value for CAD-SCD.

### The ability of specific miRNAs in distinguishing CAD-activated SCD and CAD-silent SCD

CAD-SCD victims were divided into two groups according to the apparent substrate of previous CAD activity, and there were 12 CAD-silent SCD victims and 18 CAD-activated SCD victims. The levels of miR-126-5p and miR-499a-5p were lower in CAD-activated SCD victims. Compared with the CAD-silent SCD subjects, the relative expression of miR-126-5p, miR-134-5p, and miR-499a-5p was 0.72-fold (*p* = 0.031), 1.1-fold (*p* > 0.05), and 0.46-fold (*p* = 0.020), respectively (Supplementary Figure 2), in the CAD-activated SCD group.

**Figure 3:**
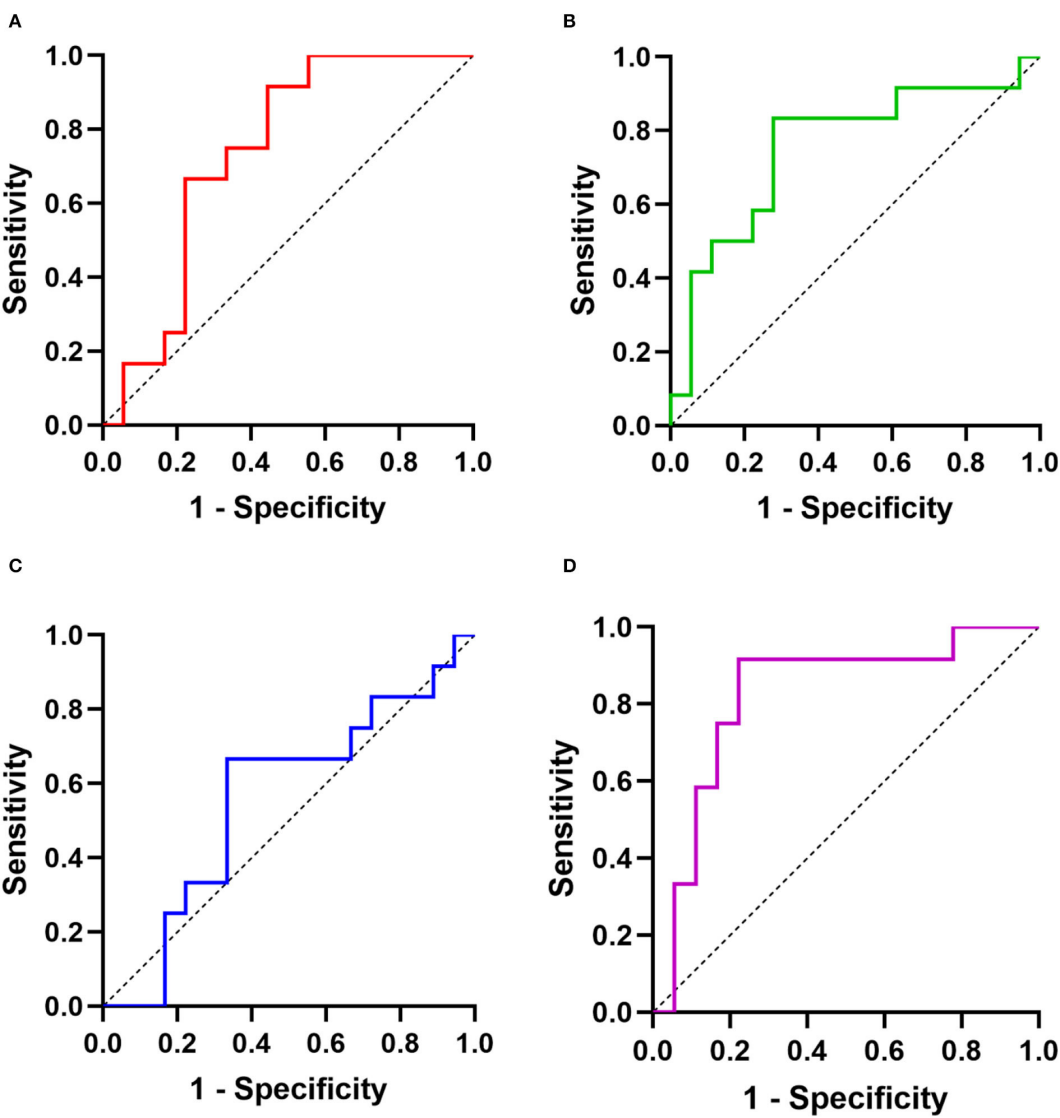
The diagnostic powers of targeted miRNAs for distinguishing CAD-activated SCD and CAD-silent SCD: ROCs of **(A)** miR-126-5p; **(B)** miR-499a-5p; **(C)** miR-134-5p; **(D)** combination (miR-126-5p and miR-499a-5p).

Next, ROC curves of targeted miRNAs were performed to estimate the diagnostic power for distinguishing old myocardial ischemia without symptoms caused by CAD in SCD victims (Figure 3). Similarly, miR-134-5p could not help the judgment (AUC = 0.56, *p* > 0.05). The AUC of miR-126-5p was 0.74 (95% CI = 0.56–0.92), showing a sensitivity of 91.7% and specificity of 55.6%. The AUC of miR-499a-5p was 0.75 (95% CI = 0.57–0.94), showing a sensitivity of 83.3% and specificity of 72.2%. This suggested that the diagnostic potencies of both miRNAs were limited. However, we analyzed the combined effect of miR-126-5p and miR-499a-5p, and found that there was a better diagnostic power of combined biomarker (AUC = 0.82, 95% CI = 0.66–0.99, sensitivity = 91.7%, specificity = 77.8%).

## Discussion

Given the high incidence of CAD-SCD, explaining these unexpected events is a significant part of daily work of clinical and forensic experts, and the crucial first step is to confirm the cause of death. Currently, identification of CAD-SCD is often dependent on the severity of coronary lesions, signs of acute heart attack, and details of death process. Due to the brief episode of ischemia before cardiac arrest, morphological evidence of heart attack and death information is often lacking, which means that CAD-SCD might be undiagnosed, or the cause of death is speculated with a potential risk of error. This study showed that cardiac miRNAs might be suitable for the diagnosis of CAD-SCD. miR-126-5p and miR-499a-5p were sensitive postmortem biomarkers of acute myocardial injury. Furthermore, they also could help differentiate whether victims had experienced occult myocardial ischemia. Although single miRNA had limited abilities in discriminating between CAD-activated SCD and CAD-silent SCD, combing the two biomarkers together could significantly improve the diagnostic power.

miRNAs had been proved to be involved in a variety of cardiac physiological and pathological processes as important regulators, including development, structural remodeling, myocardial ischemia, and heart failure. In different pathophysiological statuses, expression of miRNAs presented some characteristic changes, with potential value as novel biological markers (18). In ischemic cardiac diseases, many miRNAs had aberrant expression, participating in the impairment of coronary circulation, cardiac compensatory response, and myocardial damage. As a common regulator in the human heart, miR-1 was found to vary when myocardial injury occurred. Compared with healthy people, an upregulation of serum miR-1 level was observed in patients with myocardial infarction, and it appeared even earlier than cardiac troponin, a sensitive clinical marker of myocardial damage (19). miR-208b could regulate cardiomyocytes response to stress and significantly increased in individuals with CAD. The expression of miR-208b was reported to correlate with the severity of the disease, being used to predict the condition and prognosis of the patients (20). Furthermore, miR-133a, a heart-specific RNA molecule, was associated with coronary events, the same role as miR-1. Hence, it was also considered a potential diagnostic marker for acute myocardial ischemia, showing a massive upregulation in patients with myocardial infarction (21). Besides, miR-21, miR-150, miR-423, etc., had been successively found as biomarkers for CAD detection and prognosis (22, 23). However, the associations between CAD-related miRNAs and SCD, the most severe outcome of the disease, were still debated despite autopsy studies reporting the forensic value of several miRNAs in acute myocardial infarction victims (16). Additionally, the majority of studies focused on changes in the level of circulating miRNAs, which were mostly cardiac-derived and subsequently released into circulation. Studying changes in cardiac levels could more accurately quantify the expression of interested miRNAs in pathological conditions. The changes in cardiac miRNAs might be more sensitive than peripheral blood counterparts.

miR-126-5p plays a prominent role in keeping normal functions of coronary arteries, specifically expressing from vascular endothelial cells (24). Wang et al. (25) showed that the level of circulating miR-126 was decreased in patients with acute coronary syndrome and proposed that it might become a new diagnostic biomarker for CAD. But other scholars showed that there was an upregulation of miR-126-5p expression for myocardial infarction (26). So, the change in direction of miR-126-5p was unclear. We found that the cardiac expression of miR-126-5p was significantly decreased in CAD-SCD victims and performed well in ROC analysis. To our knowledge, this is the first study to report that miR-126-5p was related to CAD-SCD and might be a useful biomarker for clinical and forensic medicine. miR-126-5p was associated with cardiovascular homeostasis (27), and we speculated the endothelial dysfunction caused by CAD might be involved in the occurrence of SCD. Moreover, miR-126-3p had also presented potential diagnostic value for myocardial infarction, and further work could focus on the other strands of miR-126 for the diagnosis of CAD-SCD (28). It is well known that miR-499a-5p was absolutely highly expressed in cardiac tissues and showed characteristic changes in a variety of cardiovascular diseases (29). Previous studies found that when patients with CAD had acute cardiac attack the level of miR-499a-5p in peripheral blood was markedly elevated and could facilitate early diagnosis of acute myocardial ischemia (30, 31). Characterized by high sensitivity and specificity, an increasing number of studies reported that circulating miR-499a-5p had a remarkable diagnostic and prognostic value in CAD and would be an effective biomarker clinically. Notably, cardiac miR-499a-5p level presented a reverse changing trend in acute cardiac syndrome victims. Compared with normal myocardial tissue, the expression of cardiac miR-499a-5p in postmortem samples was statistically decreased in myocardial infarction decedents (16). This study demonstrated deregulation of miR-499a-5p in CAD-SCD victims, who had more severe ischemia. The result of this study was similar to the previous study. We suggested that the detection of cardiac miR-499a-5p was expected to become a reliable diagnostic approach for CAD-SCD. Previous studies indicated that miR-499a-5p was associated with cardiac function, and it might release into circulation as a result of cardiomyocyte damage (32).

Myocardial hypertrophy and scars were detected at autopsy in a proportion of CAD-SCD victims without clinically diagnostic CAD, which means that they had experienced asymptomatic heart attacks. These demonstrations of prior CAD activity had been proved to be related to the risk of SCD (33). In medico-legal examination, myocardial hypertrophy and fibrosis were typical signatures of myocardial ischemia, which could help evaluate the severity of CAD conditions. In contrast, the other part of SCD victims had a silent CAD. Lacking signs of old myocardial injury mean they were often autopsy-negative, except for coronary lesion. Hence, cardiac and forensic pathologists should pay more attention to the identification of CAD-silent SCD. This study demonstrated that miRNAs could be used as a marker to judge CAD activity, besides morphological examination. Although the capacity of single miRNA was limited in terms of discriminating activated and silent CAD, the combined biomarker showed a remarkable diagnostic power to help make more accurate evaluation of CAD condition.

This study has many limitations. First, we enrolled traumatic victims as controls to prove the diagnostic value of interested miRNAs. However, some toxicants that could affect the expression of cardiac miRNAs were not investigated in this study. Further studies could include different poisoning victims as study subjects. The miRNA quantification was blinded to the experimenters and analysts, but the cardiac macroscopic and microscopic examination could not be performed blindly by the pathologists and forensic experts, which might slightly increase the risk of detection bias. The sample size of the study population was small, making the findings to be clarified further. In addition, the clinical details of the study population were lacking, especially for some chronic diseases being related to miRNAs and SCD. For example, diabetes mellitus and, in particular, diabetic autonomic neuropathy were proved to be predictors of silent myocardial ischemic and sudden death (34), and the variants in miR-499 gene have been shown to increase the risk of cardiac autonomic neuropathy development in people with diabetics (35). This can help in having better interpretations of our findings. Unfortunately the clinical characteristics were not available because the vast majority of the subjects died suddenly in out-of-hospital settings, and the clinical data could not be obtained. So, we were unable to demonstrate this hypothesis, and these potential associations may warrant further investigations.

It can be concluded that cardiac miR-126-5p and miR-499a-5p were decreased in CAD-SCD victims, which might be novel diagnostic biomarkers for SCD caused by CAD. The expression of cardiac miR-134-5p showed no significant difference. Additionally, the combination of miR-126-5p and miR-499a-5p was a good indicator for the assessment of CAD condition, presenting an effective diagnostic power.

## Data availability statement

The raw data supporting the conclusions of this article will be made available by the authors, without undue reservation.

## Ethics statement

The studies involving human participants were reviewed and approved by the Medical Ethics Committee of Sichuan University. The patients/participants provided their written informed consent to participate in this study.

## Author contributions

LL, LY, and BC contributed to the conception and design. ML and LY contributed to the sample acquisition. LL and XH contributed to the experiments. BC supervised the study. LL performed statistical analysis and wrote the draft. All authors reviewed and revised the manuscript and approved the submitted version of the manuscript.

## Funding

This study was funded by the Sichuan Science and Technology Program (2021YFH0147).

## Conflict of interest

The authors declare that the research was conducted in the absence of any commercial or financial relationships that could be construed as a potential conflict of interest.

## Publisher's note

All claims expressed in this article are solely those of the authors and do not necessarily represent those of their affiliated organizations, or those of the publisher, the editors and the reviewers. Any product that may be evaluated in this article, or claim that may be made by its manufacturer, is not guaranteed or endorsed by the publisher.
