# Supplementary material for: Diagnostic value of cardiac miR-126-5p, miR-134-5p, and miR-499a-5p in coronary artery disease-induced sudden cardiac death
Source: Front Cardiovasc Med. 2022 Aug 25;9:944317. doi: 10.3389/fcvm.2022.944317 (PMC9457639; doi:10.3389/fcvm.2022.944317)
Supplement: Supplementary file 1 [file Data_Sheet_1.docx]

Supplementary Table 1 Characteristics of CAD-SCD and control group

|  | CAD-SCD | Fatal trauma | *p* |
| --- | --- | --- | --- |
| Age | 52.2±9.3 | 51.7±9.9 | >0.05 |
| Gender |  |  | >0.05 |
| Male | 19(63.3) | 19(63.3) |  |
| Female | 11(36.7) | 11(36.7) |  |
| Time of death |  |  | >0.05 |
| 00:00-05:59 | 7(23.3) | 3(10.0) |  |
| 06:00-11:59 | 8(26.7) | 8(26.7) |  |
| 12:00-17:59 | 5(16.7) | 10(33.3) |  |
| 18:00-23:59 | 10(33.3) | 9(30.0) |  |
| Season of death |  |  | >0.05 |
| Spring | 6(20.0) | 8(26.7) |  |
| Summer | 8(26.7) | 10(33.3) |  |
| Autumn | 4(13.3) | 7(23.3) |  |
| Winter | 12(40.0) | 5(16.7) |  |
| Single-vessel CAD | 10(33.3) | 12(40.0) | >0.05 |
| Diseased branch |  |  |  |
| LAD | 22(73.3) | 25(83.3) | >0.05 |
| LCX | 15(50.0) | 13(43.3) | >0.05 |
| RCA | 21(70.0) | 13(43.3) | 0.037 |


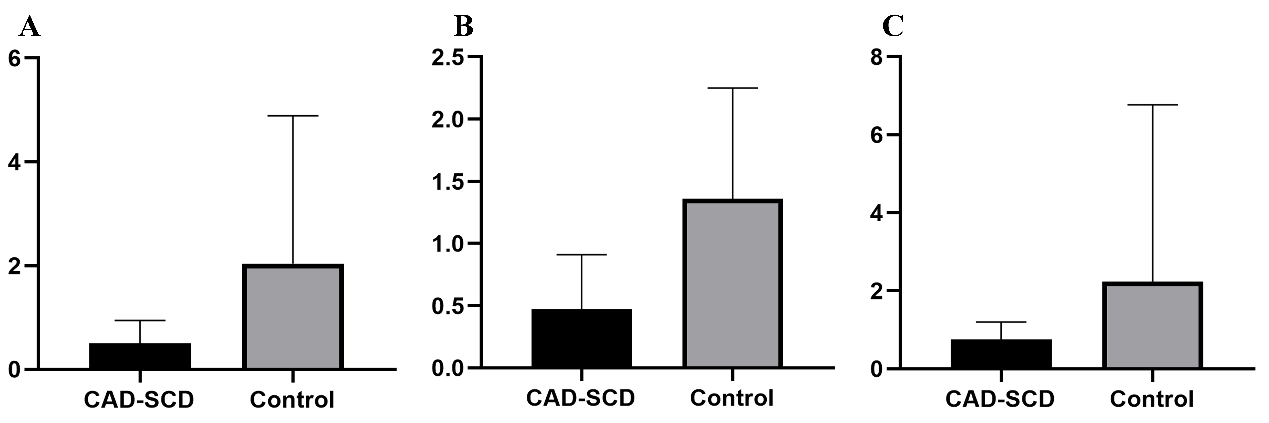


Supplementary Figure 1 The relative expression of three miRNAs in CAD-SCD and control groups. (A) miR-126-5p. (B) miR-499a-5p. (C) miR-134-5p.


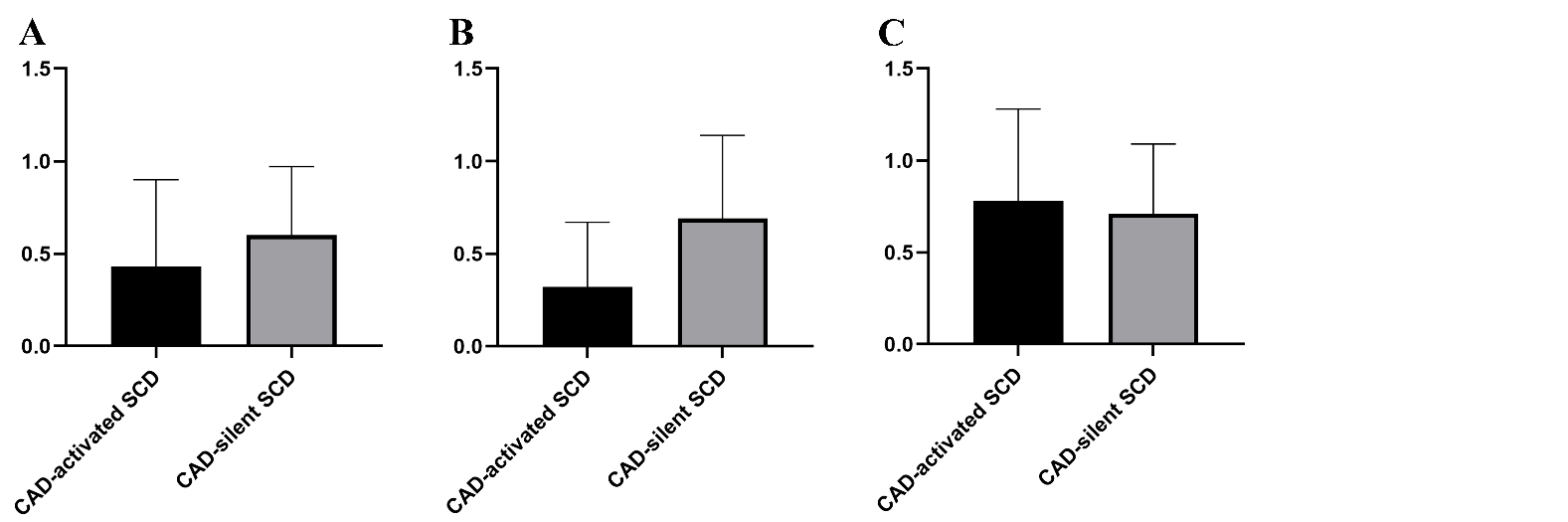


Supplementary Figure 2 The relative expression of three miRNAs in CAD-activated SCD and CAD-silent SCD groups. (A) miR-126-5p. (B) miR-499a-5p. (C) miR-134-5p.
